# Miro—Working beyond Mitochondria and Microtubules

**DOI:** 10.3390/cells7030018

**Published:** 2018-03-04

**Authors:** Bor Luen Tang

**Affiliations:** 1 Department of Biochemistry, Yong Loo Lin School of Medicine, National University of Singapore, Singapore 117597, Singapore; bchtbl@nus.edu.sg; Tel.: +65-6516-1040; 2 NUS Graduate School for Integrative Sciences and Engineering, National University of Singapore, Singapore 117597, Singapore

**Keywords:** Mitochondrial Rho (Miro), mitochondria, peroxisome, microtubules, actin

## Abstract

The small GTPase Miro is best known for its regulation of mitochondrial movement by engaging with the microtubule-based motor proteins kinesin and dynein. Very recent findings have now showed that Miro also targets peroxisomes and regulates microtubule-dependent peroxisome motility. Moreover, Miro recruits and stabilizes the myosin motor Myo19 at the mitochondria to enable actin-based mitochondria movement, which is important for mitochondrial segregation during mitosis. Miro thus has much broader functions that previously known, and these new findings may have important implications on disease pathology.

## 1. Miro, Mitochondria and Microtubules Traffic

Mitochondrial Rho (Miro) is a somewhat atypical small GTPase that is best known for functions in mitochondria transport and homeostasis [1,2,3]. Found in many (although not all) uni- and multicellular eukaryotes, the Miro orthologues harbor two GTPase-like domains that flank two Ca^2+^-coordinating EF-hands, as well as a C-terminal hydrophobic domain that allows membrane insertion. This domain structure is unusual amongst members of the small GTPase superfamily and is indicative of MIRO’s functional activities being regulated by Ca^2+^, as well as being specific to the particular sites of their membrane anchorage. Indeed, the Miro GTPases are localized to the mitochondrial outer membrane and play critical roles in intracellular mitochondria movement in metazoans, particularly over long distances along microtubule tracts in neurons [2], as well as mediating intercellular transport of mitochondria between cells via tunneling nanotubes [4]. Miro mediates bidirectional mitochondrial movement along microtubule tracts by engaging both kinesin and dynein [5], the former via the cargo adaptors of the Milton/Trak family [6,7,8,9]. In addition, Miro also has roles in mitochondrial fusion and fission dynamics through its modulation of the mitochondrial dynamin Drp1 [10] and interactions with the mitochondrial fusion proteins mitofusins 1 and 2 [11]. Miro turnover is regulated by the PTEN-induced putative kinase 1 (PINK1)/Parkin pathway [12]. PINK1s phosphorylate Miro, thus promoting its interaction with the E3 ubiquitin ligase Parkin. The latter promotes Miro ubiquitination and degradation, which effectively arrests axonal transport of damaged mitochondria [12]. On the other hand, PINK1 phosphorylated Miro also recruits Parkin to the damaged mitochondria, which then tags these for mitophagic destruction [13]. The multifaceted activity of Miro in controlling mitochondrial motility and turnover, particularly in neurons with high metabolic energy demands, is therefore pivotal for neuronal cell activities, with any functional compromise potentially leading to neurodegeneration [14].

The importance of Miro’s function in neurons can be gleaned from the phenotypes of Miro gene silencing and deletion. Silencing of the mammalian Miro paralogues, Miro1 and Miro2, in murine dorsal root ganglia (DRG) neurons resulted in altered mitochondrial distribution and disrupted axonal mitochondrial motility, respectively [11]. Mice with global deficiency of Miro1 were cyanotic and died at postnatal day zero, apparently due to defective nervous system control of respiration [14,15], but Miro2 knockout mice were viable [15]. Miro1 was shown to be the primary regulator of mitochondrial transport in both axons and dendrites, with its deletion resulted in mitochondria depletion from distal dendrites and compromised neuronal viability [15]. Notably, mice with neuron-specific conditional knockout of Miro1, although viable for a longer time, exhibited severe upper motor neuron disease symptoms with clear defects in retrograde axonal mitochondrial transport [14].

This rather exclusive view of Miro’s major function in microtubule-based mitochondrial dynamics and trafficking could now change considerably with some very recent findings. Two of these are particularly prominent, and are discussed further in the sections below. The first pertains to the demonstration of Miro’s targeting to peroxisomes and its role in peroxisome transport along microtubules [16,17]. The second concerns Miro’s recruitment of the mitochondrial actin motor Myo19 onto the outer mitochondrial membrane, thus mediating actin-based mitochondrial movement [18]. 

## 2. Miro and Peroxisomes

Peroxisomes are ubiquitously present in metazoans and have important metabolic functions, particularly fatty acid oxidation and reduction of reactive oxygen species (ROS), both of which requires coordination with metabolic processes at the mitochondria [19] and the endoplasmic reticulum. Distributed rather uniformly in mammalian cells, peroxisomes also move along microtubules via kinesin and dynein motors. Small GTPases such as RhoA [20], as well as members of the Arf and Rab families [21], have been shown to be associated with the peroxisome and have roles in the organelle’s engagement of motors and cytoskeleton. Two very recent reports have now showed that Miro1 could be localized to peroxisomes and is involved in microtubule-based peroxisome trafficking. 

Okumoto et al. [17] identified four distinct splice variants for human Miro1, which the authors termed Miro1-var1-4, and similar splice variants are also recognizable in the murine genome. The variants Miro1-var2-4 contain short 32 amino acid (aa) or 41 aa (or both) insertions between the second GTPase domain and the C-terminal membrane anchor of Miro1-var1. Fractionation studies with an antibody targeting this insertion sequence indicated that Miro1-var2 and possibly the much less abundant –var4, both containing the 32 aa insertion 1, could be found in fractions enriched with peroxisome membranes. The authors showed that insertion 1 has a peroxisomal biogenesis factor Peroxin 19 (Pex19) binding site that allows peroxisome targeting [22] of the Miro variants and membrane insertion as tail-anchored proteins. Exogenous expression of Miro1-var4 at a low level in HeLa cells induced peroxisome accumulation (but not mitochondria) at the cell periphery and increased directional movement frequency (DMF) of EGFP-labelled peroxisomes. Peroxisome DMF is conversely reduced by Miro1 silencing to a level similar to microtubule disruption by nocodazole, which could be rescued by RNAi-resistant forms of Miro1-var2 and -4, but not Miro1-var1. Furthermore, immunoprecipitation analyses showed that Miro1-var2 and -4 interact with the kinesin motor adaptor Trak2, and Miro1 variant-mediated peroxisome transport therefore likely occurs in a similar mode to that of mitochondria in terms of kinesin motor engagement. 

In another report, Castro et al. [16] also showed that Miro1 could be peroxisome-targeted via interaction with Pex19. The authors enforced Miro1 targeting to peroxisomes by conjugating it with the peroxisome targeting sequence of Peroxin 26 (Pex26), and showed that overexpression of either wild-type or a constitutively-active Miro1 GTPase mutant induced peroxisome redistribution and accumulation at the periphery of COS-7 cells without affecting mitochondria. On the other hand, over-expression of either a GTPase-deficient or an EF-hand mutant of Miro1 resulted in peroxisome clustering throughout the cytoplasm. Peroxisome-targeted Miro1 increased movement of peroxisomes in COS-7 cells and induced peroxisome proliferation in human skin fibroblasts. Interestingly, it also induced long membrane protrusions in Pex5 (a critical factor for peroxisome protein import)-deficient fibroblasts that co-localized with microtubules, suggesting that these are formed by the pulling forces generated by Miro1 and the microtubule-associated motors. The authors developed a mathematical model that recapitulated dynamic changes in peroxisome morphology resulting from the action of peroxisome membrane remodeling proteins such as Pex11β. Miro1-mediated motor forces along microtubules could thus elongate and divide peroxisomes, which impacts on peroxisome distribution and proliferation. Although details of peroxisome engagement of Miro1 and mechanistic details of how the latter regulates peroxisome movement along microtubules remain to be explored (particularly the lack of understanding in this regard on retrograde movements involving Miro and dynactin/dynein), the findings described above have extended the microtubule transport regulatory activity of Miro1 to another organelle.

## 3. Miro, Myo19 and Actin-Based Mitochondrial Movement

Not only does Miro work on another organelle, more new evidence indicates that it may also engage another cytoskeletal platform for mitochondrial movement in cells. Kittler’s laboratory has shown previously that Miro1 is the principal regulator of mitochondrial movement in neurites, and observed that Miro1 deletion leads to depletion of mitochondria from distal dendrites but not axons [15]. Furthering their work on the individual role of Miro1 and Miro2 in mitochondrial transport with mouse embryonic fibroblast (MEF) cell lines bearing either single or double knockouts (DKO) of Miro1/2, the authors made several interesting observations [18]. Firstly, both Miro1 KO and Miro2 KO MEFs showed a significant reduction in mitochondrial mobility (both short and longer range) by live imaging, as quantified by mitochondrial displacements (read as a percentage of mitochondria that changed their position over a 10 s period) compared to wild-type cells, with the DKOs showing a more drastic reduction. However, in Miro1 KO and even in DKO cells, mitochondria could still often be found aligned with microtubule filaments. In fact, although diminished, fast tubulin-dependent mitochondrial long-range transport events (quantified as directional mitochondrial displacements of at least 5 µm in distance at a velocity higher than 0.15 µm/s), could still be detected (and which was completely disrupted by vinblastine). TRAK1 and TRAK2 remained detectable in mitochondrial fractions, and form functional complexes at the outer mitochondrial membrane to facilitate anterograde mitochondrial movement, and these were possibly engaged via the mitofusins. However, TRAK2-mediated retrograde mitochondrial transport has an obligatory requirement for Miro1. Miro1 thus appears to facilitate mitochondrial retrograde transport. The Miro proteins are therefore important, but are not absolutely required for anterograde mitochondrial transport. The results also suggest that Miro may play a regulatory role in kinesin or dynein engagements rather than their direct recruitment. 

What contributed to the significant reduction in the shorter range mitochondrial displacements in Miro1/2 KOs and DKOs? The authors explored the possibility of a loss in actin engagement, particularly through the mitochondrial localized actin motor Myo19 [23,24]. Interestingly, endogenous Myo19 levels were reduced in Miro2 KO cells and more drastically so in the DKOs. Loss of either Miro1 or Miro2 diminished Myo19 levels in the cells’ mitochondrial fraction, and the latter is almost completely lost from DKO mitochondria. Imaging analyses indicated that the loss of Myo19 from mitochondria could be rescued by the over-expression of either Miro1 or 2, but not by their mutants lacking a mitochondrial targeting signal. Miro1 and Miro2 form complexes with Myo19, recruit the latter to the mitochondria and stabilize it at the mitochondrial membrane. The authors further showed that loss of Miro resulted in uneven segregation of mitochondria during cell division and a reduced mitosis rate, which is only partially rescued by over-expression of Myo19. Mitochondrial segregation during mitosis could thus involve coordination of both tubulin and actin-based motors by Miro. That both a microtubule-based and an actin-based motor could be engaged by a single small GTPase has precedence in the cases of the Rab family members Rab10 [25,26] and Rab11 [27,28]. This duality of microtubule/actin motor engagements, however, is the first demonstration for a small GTPase that is responsible for mitochondrial transport. 

## 4. New Perspectives in Miro Function and Their Implications

The recent findings on Miro briefly described above have important implications. Firstly, the peroxisome targeting and function of Miro1 splice variants indicate yet another layer of functional and perhaps also evolutionary links between peroxisomes and mitochondria. Initially thought to arise exclusively from the ER membrane based on studies in yeast [29], McBride’s group has recently shown instead that nascent peroxisomes in human fibroblasts are hybrids of both mitochondrial and ER-derived pre-peroxisomes [30]. Based on this finding and that peroxisome activities of fatty acid oxidation and ROS reduction are connected to apparently upstream processes in the mitochondria, Speijer [31] has recently hypothesize that peroxisomes are inventive adaptations resulting from (or evolved subsequent to) the establishment of the endosymbiotic partnership in eukaryogenesis. This is an interesting notion and how mitochondrial and peroxisome functional (or even physical) interactions could be coordinated by Miro within the cell would be an interesting question to further pursue. 

**Figure 1:**
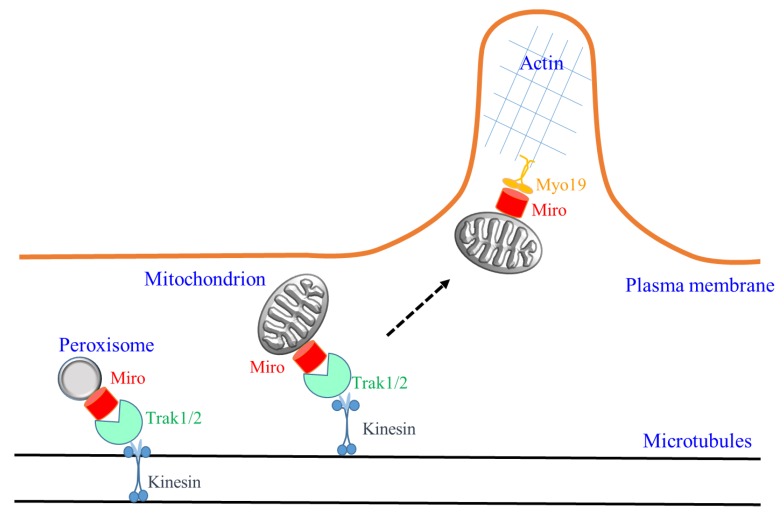
A schematic illustration of the new findings on Miro activity discussed in the text. Miro (splice isoforms not specified) could be localized to both mitochondria and peroxisomes to mediate microtubule-based transport by engaging the kinesin adaptors Trak1/2. Miro could also engage the actin motor Myo19 for actin-based mitochondrial transport. In neuronal cells, a switch from long-range Miro-mediated microtubule-based transport along axons and dendritic shafts to short-range actin-based transport in dendritic spines and growth cones may be conceivable, as illustrated speculatively by the dotted arrow.

It now appears that cellular mitochondria movements are dependent on both microtubule- as well as actin-based motors via Miro’s engagement of either Trak1/2 or Myo19. The relative importance of these two modes in different cell types requires further clarification, as Myo19-action-based movements in mitochondria partitioning would be more important for dividing cells as opposed to terminally differentiated, non-dividing cells. However, even within the same cell, there could be a division of labor with differential engagement of microtubule or actin-based motor by Miro. With neurons, for example, the need for long-range mitochondria transport in axons and dendritic shafts would require TRAK-microtubule based transport. However, actin-based, short-range movements would become important in specialized structures like axonal growth cones and dendritic spines (see Figure 1). In this regard, how Miro could switch mitochondrial transport from one cytoskeletal track to another would be another interesting question to tackle. Along the same line of thought, whether peroxisomes could also be transported by action-based motors via Miro1, in both neuronal and non-neuronal cell types, would also be worth exploring. 

Another possibility arising from the new findings is that Miro-dependent organelle transport dysfunction-induced disease states would have contributions from both mitochondrial and peroxisomal functional deficits, in fact likely with both deficits acting in synergy. Mitochondrial functional deficiencies underlie many neurodegenerative disorders [32]. As previously shown, neuron-specific Miro1-knockout mice with defects in movement and distribution of mitochondria induce a motor neuron disease-like phenotype [14]. Interestingly, it is notable that defects in peroxisomal biosynthesis of Zellweger Syndrome resulted in neurodevelopmental defects, as well as neurodegeneration [33]. Peroxisome deficiency in neural cells of mice arising from conditional knockout of Pex5 causes severe demyelination, axonal degeneration and neuroinflammation [34]. Microtubule-dependent peroxisome movement is found to be impaired in cells with spastin gene (*SPAST*) mutations from patients with Hereditary Spastic Paraplegia [35]. Deficiency in the peroxisome fatty acid β-oxidation multifunctional protein-2 (MFP2) causes cerebellar ataxia resulting from Purkinje cell axonal dystrophy [36]. The above non-exhaustive list of examples illustrates the importance in understanding Miro activity and its potential dysfunction that may lead to combinatorial mitochondrial and peroxisome defects in diseases.
